# Performance and Stress Analysis of Metal Oxide Films for CMOS-Integrated Gas Sensors

**DOI:** 10.3390/s150407206

**Published:** 2015-03-25

**Authors:** Lado Filipovic, Siegfried Selberherr

**Affiliations:** Institute for Microelectronics, Technische Universität Wien, Guβhausstraβe 27-29/E360 Wien, Austria; E-Mail: Filipovic@iue.tuwien.ac.at

**Keywords:** metal oxide, gas sensor, SnO_2_, spray pyrolysis, ZnO, indium-tin-oxide, In_2_O_3_, intrinsic stress, thermo-mechanical stress, simulation

## Abstract

The integration of gas sensor components into smart phones, tablets and wrist watches will revolutionize the environmental health and safety industry by providing individuals the ability to detect harmful chemicals and pollutants in the environment using always-on hand-held or wearable devices. Metal oxide gas sensors rely on changes in their electrical conductance due to the interaction of the oxide with a surrounding gas. These sensors have been extensively studied in the hopes that they will provide full gas sensing functionality with CMOS integrability. The performance of several metal oxide materials, such as tin oxide (SnO_2_), zinc oxide (ZnO), indium oxide (In_2_O_3_) and indium-tin-oxide (ITO), are studied for the detection of various harmful or toxic cases. Due to the need for these films to be heated to temperatures between 250 °C and 550 °C during operation in order to increase their sensing functionality, a considerable degradation of the film can result. The stress generation during thin film deposition and the thermo-mechanical stress that arises during post-deposition cooling is analyzed through simulations. A tin oxide thin film is deposited using the efficient and economical spray pyrolysis technique, which involves three steps: the atomization of the precursor solution, the transport of the aerosol droplets towards the wafer and the decomposition of the precursor at or near the substrate resulting in film growth. The details of this technique and a simulation methodology are presented. The dependence of the deposition technique on the sensor performance is also discussed.

## 1. Introduction

**Figure 1:**
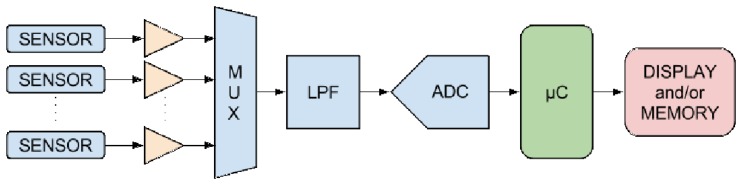
Sensor array with interface electronics blocks, which include the amplifiers, multiplexer (MUX), low-pass filter (LPF), analog to digital converter (ADC), microcontroller (μC) and display and/or memory.

**Figure 2:**
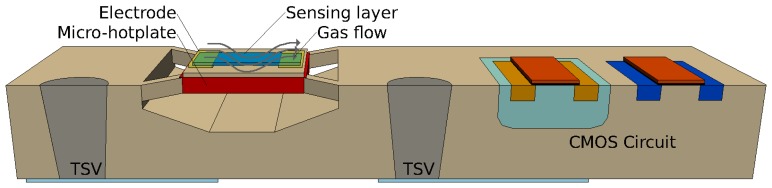
Setup of the integrated gas sensor as a system-on-chip. TSV, through-silicon via.

The ability to detect toxic and harmful gases in our environment through hand-held and wearable devices is a subject of extensive research. Recent discoveries in the use of metal oxides as gas sensing materials are at the forefront for enabling significant progress in moving away from bulky sensor architectures [1–10]. The miniaturization of electronic devices has proven to be essential, while the gas sensor field is still lagging behind the overall progress of CMOS and MEMS devices. Two materials have been proven to exhibit all of the properties required for a good gas sensing performance, namely zinc oxide (ZnO) [11–14] and tin oxide (SnO_2_) [15–17], while others, such as indium tin oxide (ITO), In_2_O_3_, CdO, ZnSnO_4_, NiO, *etc.*, have also been widely studied [13]. However, before true integration of gas sensor components inside gadgets, such as smart phones and wrist watches, can be achieved, several challenges must still be overcome:

- Until recently, available gas sensors relied on a bulky architecture whose manufacturing was not compatible with that of a conventional CMOS process sequence. The integration with CMOS processing is essential in order to combine the sensor with MEMS and CMOS microelectronics. Currently, the deposition of metal oxide materials is being performed using several techniques, such as chemical vapor deposition [18], sputtering [19], pulsed-laser deposition [20], the sol-gel process [21] and spray pyrolysis [15].
- Thin metal-oxide layers can only act as gas sensors when heated to temperatures between 250 °C and 550 °C, which means that a micro-hotplate must accompany each sensor. The integration of the hotplate and the sensor with the required analog and digital circuitry, as shown in Figure 1, is essential. A sensor array is needed in order to enable the detection of various gases in the environment using a single sensor unit. Metal oxides generally react to several potential toxins; therefore, the implementation of the sensor array, where each sensing gas is detected independently, is crucial for the design of a useful product.
- Three-dimensional integration with through-silicon vias (TSVs) can be used to connect the various parts of the sensor circuit, while avoiding the signal loss and delay associated with long metal routing. Figure 2 shows a potential integrated gas sensor as a system-on-chip [22], where the TSVs are used to carry the signal to the next parts of the circuit, such as the amplifier and multiplexer (MUX) shown in Figure 1.

As already depicted in Figure 1, the operation of a smart gas sensor implies the ability to detect a multitude of hazardous gases in the environment, for which multiple sensor circuits are required. The analog signal from these sensors is passed through an amplifier to a multiplexer. The output from the multiplexer is sent through a low-pass filter (LPF) and an analog to digital converter (ADC) before the signal can be analyzed with a microcontroller (μC) and eventually displayed or stored [8]. Although the major part of the electronics consists of analog and digital CMOS circuitry, the most complex component, for integration and manufacturing, is the sensor itself.

During deposition of the metal oxide thin films, stress builds up, which can have negative effects on the device reliability. The post-processing stress in a thin film is a result of two stress components: the intrinsic stress, which arises during the film growth, and the thermo-mechanical stress, which is a result of the difference in the deposition temperature and the subsequent cooling to room temperature. The thermo-mechanical stress is a concern, when high temperatures are used for the metal oxide deposition due to the difference in the coefficients of thermal expansion (CTE) between the depositing material and the silicon substrate. The growth mode of thin films depends on the surface free energy of the deposit, the substrate and the interface between the two materials. The stress evolution during the deposition of tin oxide, zinc oxide, indium oxide and indium-tin-oxide films is analyzed in Section 3. The growth process is characterized by the Volmer–Weber growth mode [23,24], where small islands of the depositing film form on the surface, which, through expansion, impinge on each other, eventually forming a coalesced thin film. Due to the surface interaction between the materials and the impingement of the depositing islands, a stress forms in the thin film [25].

## 2. Metal Oxide Gas Sensor Performance

**Figure 3:**
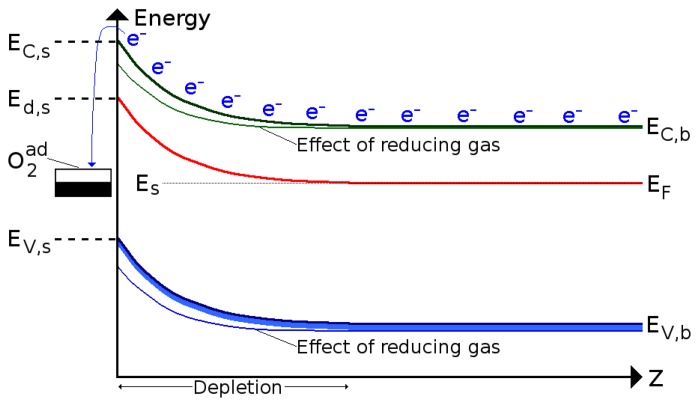
Schematic representation of the band bending effect caused by oxygen adsorption and subsequent introduction of a reducing gas.

Metal oxide materials can be used as gas sensors, when heated to temperatures between 250 °C and 550 °C. At these temperatures, oxygen is adsorbed at the metal oxide surface by trapping electrons from the bulk material. The result is an overall decrease or increase in the metal oxide resistance, depending on whether the material is n-type or p-type, respectively. The band bending at the metal oxide/ambient interface is depicted in Figure 3. The introduction of a target gas in the atmosphere causes a reaction with the oxygen, removing it from the interface and reducing the band bending effect and, thereby, the overall resistance [26].

**Figure 4:**
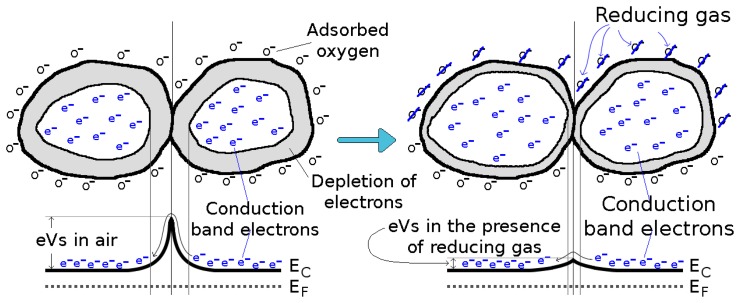
Gas sensing function and conduction mechanism for a porous metal oxide, where the oxygen and reducing gas can penetrate to interact with each grain.

**Figure 5:**
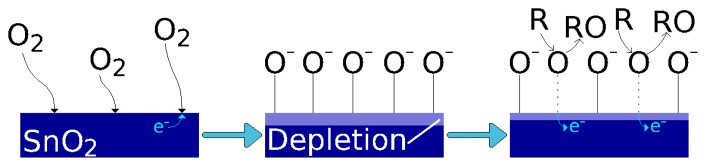
Gas sensing function for a compact tin oxide film. The reaction occurs only at the top surface of the deposited tin oxide. The symbol R refers to a reducing gas.

The thickness of the depletion layer is in the order of the Debye length, defined as:

(1) $$\lambda_{D}=\sqrt{\frac{\epsilon_{0}k_{B}T}{q^{2}n_{C}}}$$

where *ϵ*_0_ is the free space permittivity, *q* is the elementary charge and *n_C_* is the carrier charge density The structure of the sensing layer will influence the sensing process. When a porous layer is deposited, the gas species can penetrate into the metal oxide to react at the surface of the grains and the grain boundaries [7], as shown in Figure 4. However, for compact metal oxide films, such as the ones studied here, the oxygen adsorption and reaction with a reducing gas occurs on the surface [26], as depicted in Figure 5.

Even though plenty of effort has been directed towards understanding the gas sensing function of metal oxide materials, briefly addressed in Figures 4 and 5, the exact chemistry of the sensing process is complex and not yet exhaustively understood [27]. A porous film is more complex to deposit when compared to a compact thin film, usually involving a sol-gel technique followed by a gelation step [27]. A compact metal oxide thin film can be deposited using a variety of techniques, including spray pyrolysis, which has recently gained traction due to its cost-effectiveness and integration within a standard CMOS processing sequence. This study concerns itself with compact metal oxide thin films, and this section discusses the gas sensing capabilities achieved by various scientific research groups.

### 2.1. ZnO Gas Sensing Capability

**Figure 6:**
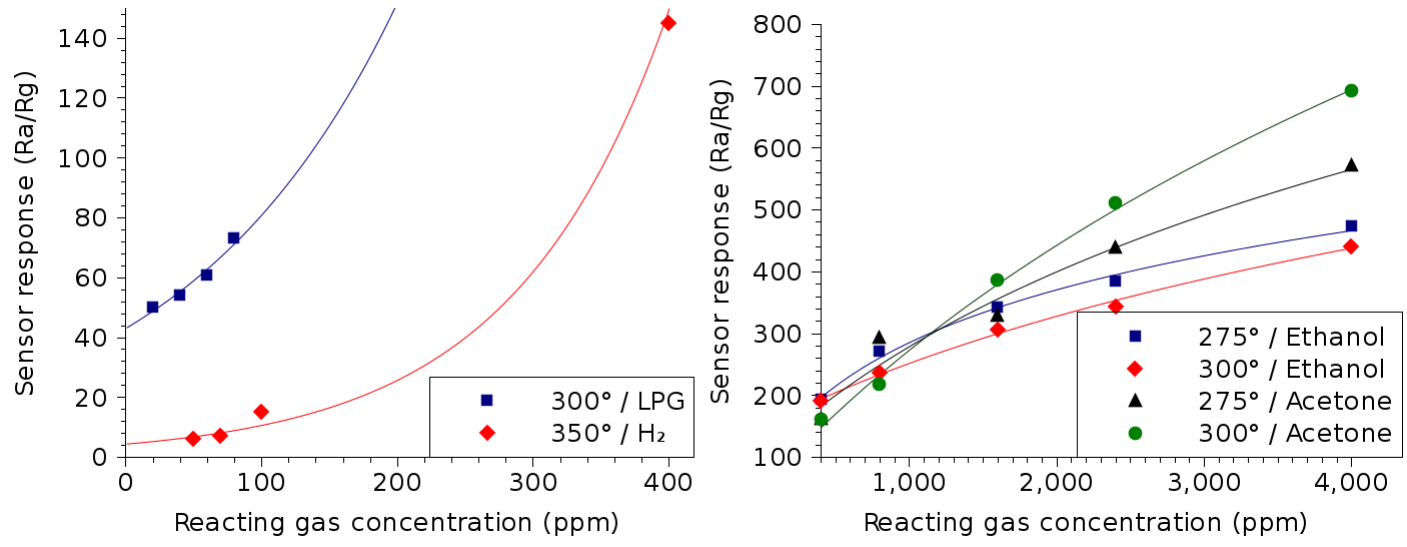
ZnO thin film sensor response dependence on the concentrations of various reacting gases. The temperatures used during measurement correspond to the optimal temperature of operation for the detection of the particular gas.

The capabilities of zinc oxide (ZnO) to detect hydrogen (H_2_), liquid petroleum gas (LPG), acetone ((CH_3_)_2_CO) and ethanol (C_2_H_5_OH) in the environment has been examined in the literature [28–30]. Acetone and ethanol belong to a group of organic chemicals referred to as volatile organic compounds (VOCs), which have a high vapor pressure at room temperature and often mix with interfering gases [31]. The sensor response is calculated by observing the resistance of the metal oxide layer in air (R*_a_*) and dividing it by the resistance measured in the presence of the desired reacting gas (R*_g_*). The temperature at which the sensitivities to ambient gases is examined reflects the peak temperature of operation for each thin film in the detection of the desired gas. The symbols given in Figure 6 represent the measured sensor responses, while the solid lines are best-fit lines, whose equations are given in Table 1.

From Figure 6 and Table 1, it is evident that ZnO can be used for the detection of several potentially dangerous gases, such as LPG, hydrogen, ethanol and acetone. For the detection of ambient hydrogen, an exponential behavior is noted, similar to the detection of LPG. The exponential response to the presence of a reacting gas is common during the early stages of detection, when the gas concentration is relatively low (<400 ppm). When the right side of Figure 6 is observed, a logarithmic dependence on the reacting gas concentration is noted. This is common when the concentration of the reacting gas is sufficiently high and moving towards saturation with respect to the ability to detect small variations.

From the acetone response in Figure 6, it is evident that below a concentration of about 1200 ppm, the response is stronger at an operating temperature of 275 °C, while above this concentration, 300 °C is preferable. This may be due to the fact that the sensing mechanism of acetone by metal oxides involves physisorption followed by chemisorption and electron transfer [32]. During physisorption, a surface acetone transfers to its isomer, which reacts with a second acetone to yield mesityl oxide; chemisorption and electron transfer can only take place thereafter [32]. When the concentration of acetone is low (below 1200 ppm), physisorption is the limiting step in the sensing mechanism, since two acetone molecules are required to generate mesityl oxide. Given that at this acetone concentration, a temperature of 275 °C is preferable, as shown in Figure 6, it can be deduced that the physisorption mechanism prefers a 275 °C operating temperature. On the other hand, chemisorption is the limiting step at acetone concentrations above 1200 ppm, which appears to prefer the higher 300 °C temperature.

### 2.2. In_2_O_3_ Gas Sensing Capability

**Figure 7:**
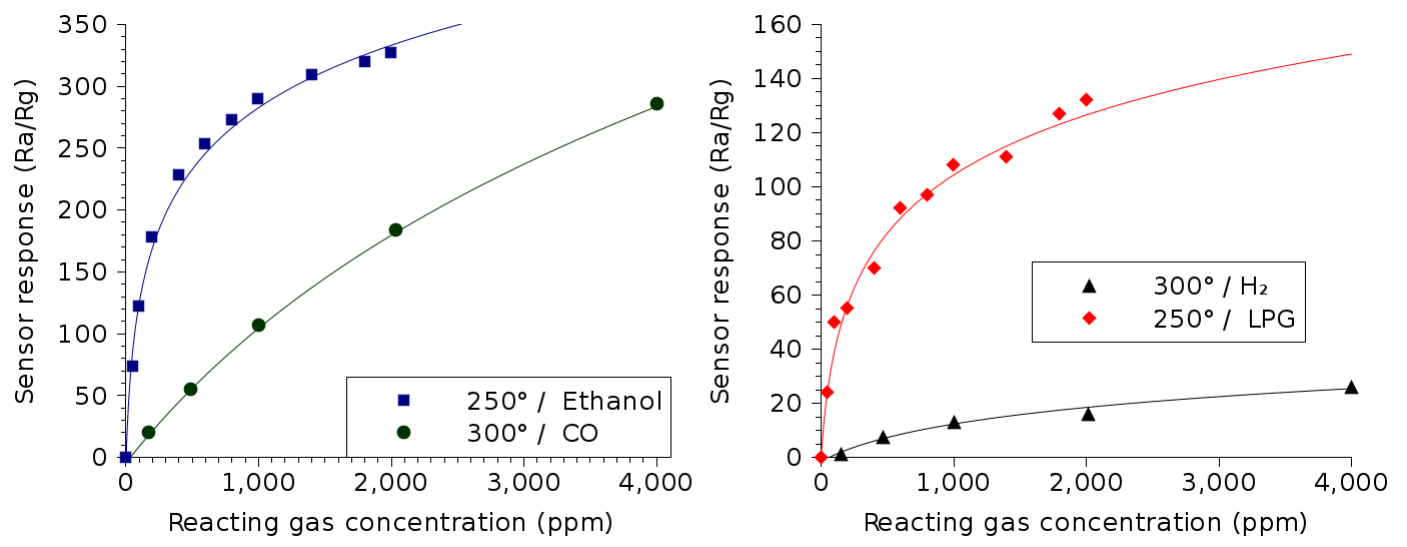
In_2_O_3_ thin film sensor response dependence on the concentrations of various reacting gases. The temperatures used during measurement correspond to the optimal temperature of operation for the detection of the particular gas.

In addition to ethanol [33], LPG [33] and hydrogen [34], In_2_O_3_ thin films have been used to detect the presence of carbon monoxide (CO) down to 150 ppm [34]. The sensing capabilities of In_2_O_3_ thin films are shown in Figure 7. Once again, the symbols refer to the measured sensor response, while the solid lines are the lines of best fits, whose equations are given in Table 2.

Similar to ZnO thin films, the overall trend for an In_2_O_3_ sensor is a logarithmic dependence on the reacting gas concentration and eventual saturation. For example, when the sensing response to ethanol and LPG is observed, it is clear that the sensing efficiency is highest with the gas concentration below about 1000 ppm. It can similarly be observed that the response to ambient hydrogen is weaker than to other reactive gases shown and that the ZnO film from Figure 6 is better suited for this particular function.

The In_2_O_3_ thin film responds very well to the presence of poisonous carbon monoxide in the environment. The response does not saturate even when 4000 ppm of CO is found in the sample ambient air. The film sensitivity appears almost linear, making for an almost ideal CO sensor, which is an essential part of every household, especially considering the odorless and colorless nature of CO gas.

### 2.3. ITO Gas Sensing Capability

**Figure 8:**
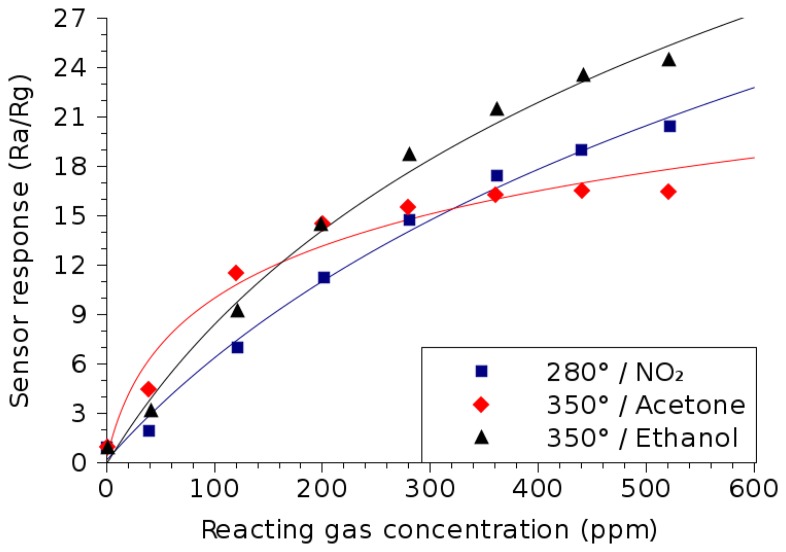
ITO thin film sensor response dependence on the concentrations of various reacting gases. The temperatures used during measurement correspond to the optimal temperature of operation for the detection of the particular gas.

Indium-tin-oxide has frequently displayed its capability as a detector of ethanol [35], acetone [35] and methanol [31], detections of which frequently feature in ongoing attempts towards the development of the electronic nose [36]. In fact, some studies suggest that ITO thin films may offer a sufficiently strong detection of alcohol to allow operation even at room temperature [31]. However, here, we concentrate on sensor operation at elevated temperatures. The ITO thin film sensing response to the presence of acetone, ethanol and nitrogen dioxide (NO_2_), a harmful chemical when inhaled even at low concentrations, is shown in Figure 8.

The sensor response shown in Figure 8 mimics what was already seen in ZnO and In_2_O_3_ thin films. The general trend is a logarithmic dependence on the reactive gas concentration; the best-fit equations that govern this trend are given in Table 3. Figure 8 shows that the response to acetone saturates much sooner than the response to ethanol or NO_2_ presence, which appear to be detectable up to and potentially beyond 500 ppm.

### 2.4. SnO_2_ Gas Sensing Capability

**Figure 9:**
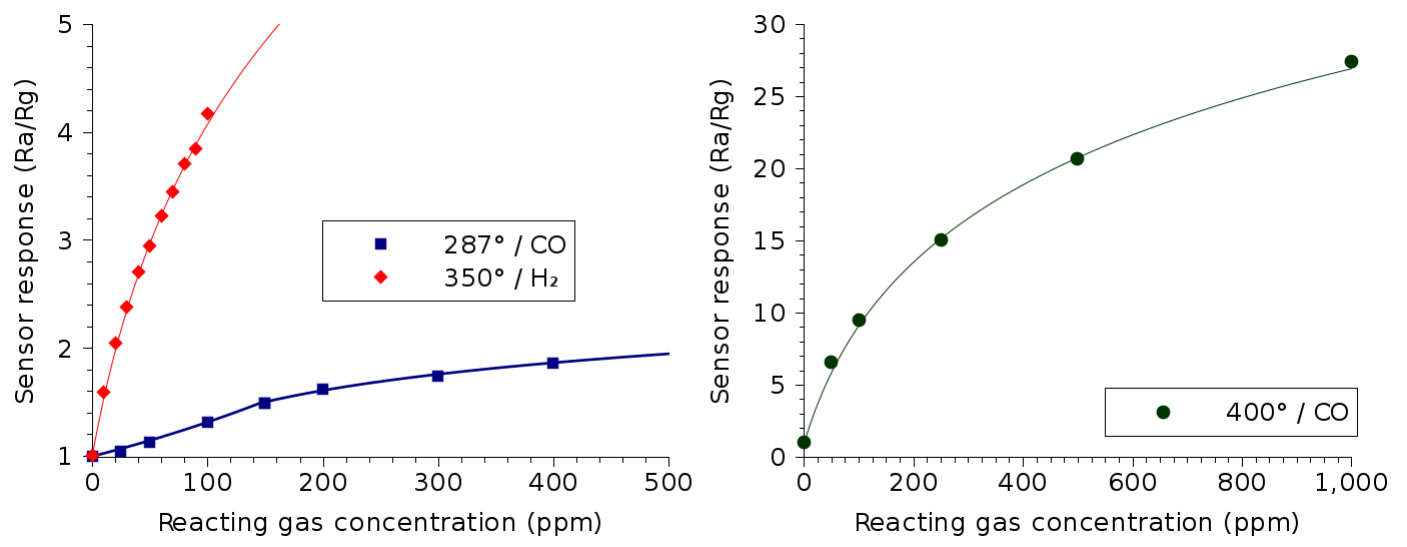
SnO_2_ thin film sensor response dependence on the concentrations of various reacting gases. The temperatures used during measurement correspond to the optimal temperature of operation for the detection of the particular gas.

The gas sensing capability of SnO_2_ is well known, and it is the most common metal oxide used for the detection of harmful gases [37]. This is in part due to its sensitivity to a broad range of gases, but also due to the ability of the material to be deposited on a silicon substrate using a variety of simple and inexpensive methods. Figure 9 shows the response of a SnO_2_ thin film to the presence of hydrogen [16] and carbon monoxide (278 °C [38], 400 °C [39]) in the environment.

The SnO_2_ sensor response to the presence of CO shown in the left plot in Figure 9 has been measured at 287 °C for a sputtered film [38]. The CO response shown in the plot on the right has been measured at 400 °C for a film, which was deposited using a spray and includes impurities in the form of Pt nanoparticles [39]. The ability to deposit SnO_2_ films using a spray pyrolysis technique is further discussed in Section 4. The effect of the Pt impurity and higher operating temperature is noted by an increase in the sensing performance by almost ten times. Once again, the symbols in Figure 9 represent measured data, while the solid lines correspond to best-fit lines, the equations of which are given in Table 4. Another interesting observation with the sensing of CO at 287 °C is that the sensor response behavior switches from exponential below 150 ppm to logarithmic above 150 ppm; this is similar to the behavior noted in the ZnO film in Section 2.1.

From Figure 9, the SnO_2_ film displays the ability to detect hydrogen at very low concentrations. While the hydrogen detection of ZnO films shown in Figure 6 appears to only start at a concentration of 100 ppm, SnO_2_ films can detect hydrogen down to 10 ppm [16].

## 3. Stress Evolution during Metal Oxide Deposition

**Figure 10:**
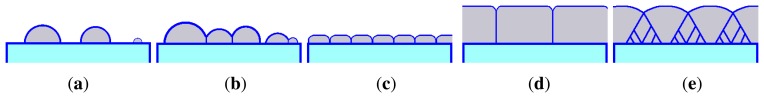
Steps during film formation using the Volmer–Weber growth mode. (**a**) 1-nucleation; (**b**) 2-impingement; (**c**) 3-coalescence; (**d**) 4a-columnar thickening; (**e**) 4b-polycrystalline thickening.

During the deposition of metals and metal oxides on oxidized silicon surfaces, the film deposits in the form of islands, which coalesce and then grow to form larger grains, a process known as the Volmer–Weber growth mode [24,25]. The intrinsic stress generated during thin film growth develops during deposition and can go through stages of compressive and tensile stresses depending on the film properties. Figure 10 summarizes the main steps involved in the film growth, as characterized in [25].

**Figure 11:**
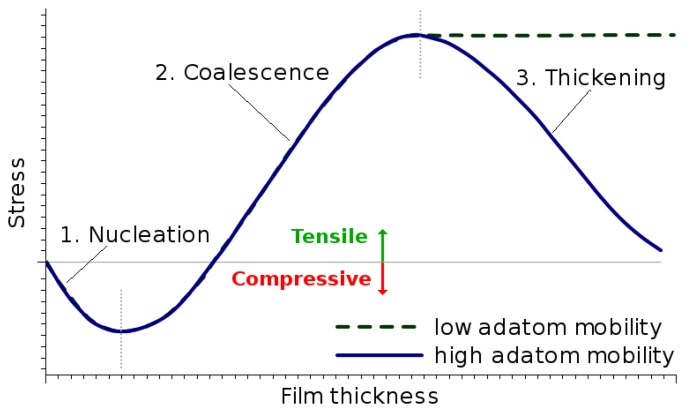
Stress evolution during the growth of metal and metal oxide films.

The initial stage is the island nucleation or the formation of small islands on the surface. As the islands grow, they impinge on each other, which generates tensile stress in the islands. When all of the islands on the surface are connected, a film is said to have coalesced. The next stage of growth is the film thickening, which can be either columnar or polycrystalline. The columnar thickening mode shown in Figure 10d refers to the growth of films that have a high adatom mobility or low melting temperatures, such as silver (Ag), copper (Cu) and aluminum (Al). The polycrystalline growth shown in Figure 10e refers to the growth of films that have a low adatom mobility or high melting temperatures, such as tungsten (W), chromium (Cr) and tantalum (Ta). Before film coalescence, there are two main stress components to consider, namely the compressive stress, which is generated due to the island nucleation, and the tensile stress generated in the thin film due to island impingement. The evolution of the stress during the growth of the two types of materials is shown in Figure 11. The generation of compressive stresses during nucleation and thickening, as well as the generation of a tensile stress during coalescence is depicted. The intrinsic stress of materials with low adatom mobility does not vary after coalescence and remains constant during thickening. However, materials with high adatom mobility experience a reduced stress, or increased compressive stress, during post-coalescence film growth.

**Figure 12:**
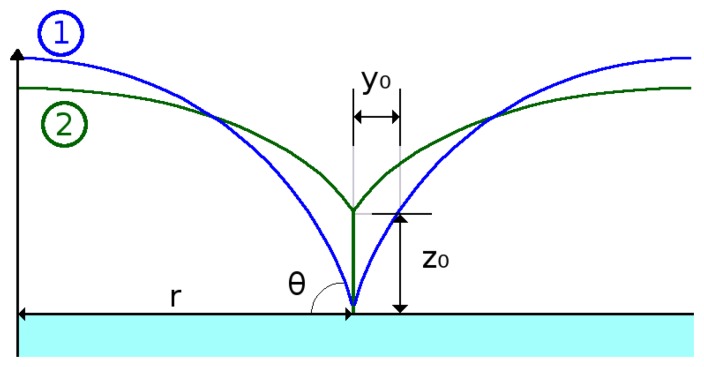
From Position 1 to Position 2, two islands impinge, resulting in a grain boundary with height *z*_0_.

During the formation and subsequent radial expansion of an island during film growth, a compressive stress builds up due to an excess in surface energy. The model that governs the build-up of compressive stress in relation to the growth of an island is given in [25] as:

(2) $$\sigma_{\text{compressive}}=-\frac{2f}{r}\cdot \frac{\sin\theta }{\left(1-\cos\theta \right)\left(2+\cos\theta \right)}$$

where *f* = *γ_gb_* − 2*γ_s_* is the surface stress (*γ_gb_* is the grain boundary energy and *γ_s_* is the surface energy), *r* is the island radius and *θ* is the contact angle between the island surface and the substrate, depicted in Figure 12. Upon island impingement, a grain boundary is formed between two islands, which results in a part of the free surface of each island being eliminated and in a significant energy reduction. This process of zipping at the grain boundary to a specific height generates tensile stress in the grains. Figure 12 shows the process of two islands impinging on each other and the formation of a grain boundary. When two islands approach each other and each attempts to increase its radius, a grain boundary forms. The generated tensile stress depends on the resulting geometry of that process, given in [25] as:

(3) $$\sigma_{\text{tensible}}=\frac{1}{2}\cdot \frac{E}{1-r^{2}}{\left(\frac{y_{0}}{r}\right)}^{1.3892}$$

where *E* is the Young's modulus and *r* and *y*_0_ are geometric parameters given in Figure 12. When two islands come together (Step 1 in Figure 12) and they each attempt to grow, each island will be hindered by the presence of the adjacent island. Therefore, instead of freely expanding its circumference, a vertical boundary will form between them, with a height *z*_0_ (Step 2 in Figure 12).

The tensile stress described by Equation (3) can relax through the transport of matter to the strained region within the grain boundary. An equation that governs this mechanism is given in [25], and more details regarding the relaxation process can be found there:

(4) $$\dot{\sigma }=-\frac{C_{o}}{t}\cdot \sigma_{\text{tensile}}\cdot e^{-Q/kT}$$

where *C_o_* is a material-dependent parameter, *h* is the film thickness, *Q* is the activation energy for the material diffusion, *k* is the Boltzmann constant and *T* is temperature. For simplicity, the relaxation phenomena will be neglected here.

### 3.1. Metal Oxide Material Properties

The material properties required for the simulation of the post-processing intrinsic stress for metal oxides of interest are given in Table 5. The properties have been collected from a literature survey of [37,40–56]. It is important to note that the intrinsic stresses listed in Table 5 correspond to a compressive stress.

### 3.2. Intrinsic Stress Generation during Metal Oxide Deposition

**Figure 13:**
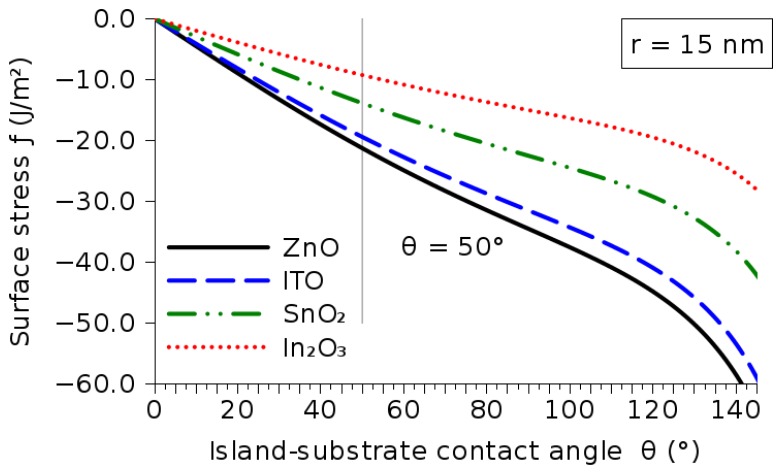
Relationship between the surface stress *f* (J/m^2^) and contact angle *θ* (°) during the deposition of the metal oxides ZnO, In_2_O_3_, ITO and SnO_2_.

The build-up of compressive stress during deposition is described by Equation (2). The stress depends on the contact angle between the island surface and the substrate *θ*, the radius of the growing islands *r* and the surface stress *f*. The island radius *r* can be measured experimentally, and some published values are listed in Table 5. The expected compressive intrinsic stresses are also listed there; this allows an estimation of the expected surface stress for the metal oxides and an analysis of the dependence on the contact angle. Figure 13 shows the relationship between the surface stress and the contact angle for the metal oxides discussed in this manuscript. From Figure 13, it is evident that the surface stress decreases as the angle increases. At a sample angle of 50°, the surface stress for the metal oxides is given in Table 6.

Although most grown metal oxides exhibit compressive stresses in the GPa range, some authors have presented methods by which the stress can be reduced to several hundred MPa in the tensile regime. This reduction is mainly achieved by varying the process parameters during sputter deposition: an increase in the amount of oxygen in the environment for SnO_2_ films [51] and an increased pressure for ITO films [57] are examples. The resulting intrinsic stresses for the SnO_2_ and ITO films are 200 MPa and 300 MPa, respectively. Another manner by which the stress becomes more tensile is by annealing or material cooling after deposition at an increased temperature [57].

### 3.3. Thermo-Mechanical Stress during Metal Oxide Cooling

**Figure 14:**
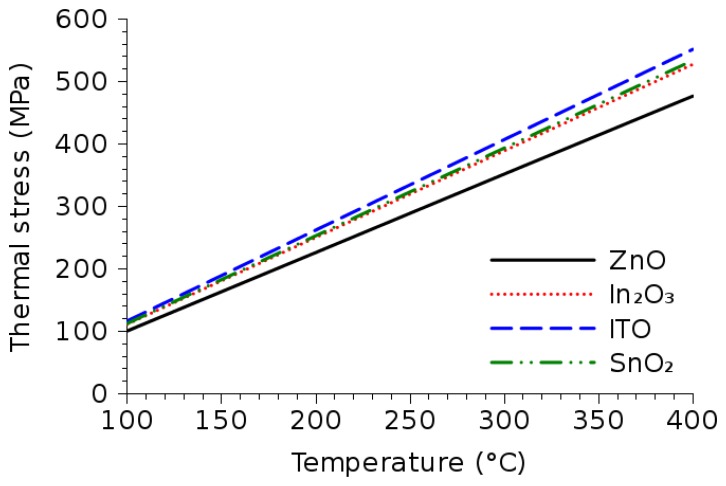
Thermo-mechanical stress for the metal oxide materials introduced in this study and its dependence on the process temperature. A linear dependence on temperature is evident. The metal oxide with the highest CTE (ITO) experiences the largest thermo-mechanical stress. Similarly, the metal oxide with the lowest CTE (ZnO) experiences the lowest stress.

The amount of tensile stress generated due to heating depends on the CTEs of the materials involved. Using the values from Table 5 and assuming a deposition on a flat silicon wafer with a 500 nm-thick SiO_2_ isolation layer, the thermo-mechanical stresses are plotted in Figure 14; the values are derived using simulations with finite element programs. The temperature refers to the deposition or annealing temperature of the wafer prior to cooling to room temperature. The CTEs of silicon and SiO_2_ are 2.6 × 10^−6^ K^−1^ and 0.5 × 10^−6^ K^−1^, respectively.

When a temperature drop Δ*T* is imposed on a material, such as is the case during cooling to room temperature after a thermal deposition step, the material experiences a strain:

(5) ϵ=α⋅ΔT

where *α* is the CTE. Therefore, the resulting stress is linear with respect to the applied temperature, as is observed in Figure 14. In addition, a larger CTE results in larger stress, which is also shown in Figure 14, where the metal oxide with the highest CTE (ITO) also experiences the highest thermo-mechanical stress. Similarly, the lowest CTE material (ZnO) experiences the lowest stress.

## 4. Spray Pyrolysis Deposition of SnO_2_

**Figure 15:**
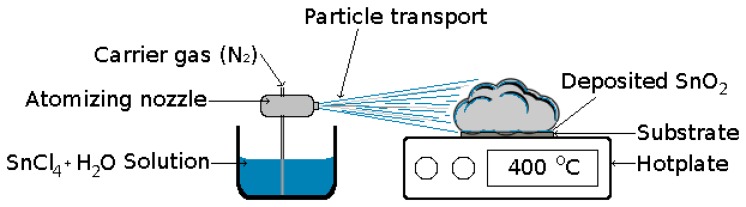
Schematic of the experimental spray pyrolysis deposition process as set up for SnO_2_ deposition.

The deposition of SnO_2_ has been performed using various techniques, such as CVD [18], sputtering [19], pulsed-laser [20], sol-gel [21] and spray pyrolysis [15]. The spray pyrolysis technique has gained traction over its alternatives due to its cost effectiveness and ease of integration in the standard CMOS process. During deposition, a gas pressure nozzle is used to atomize a SnCl_4_+H_2_O solution. The nozzle generates very small droplets, which are directed towards the substrate, where SnO_2_ is deposited on top of a heated wafer, as shown in Figure 15. Using this method, substrates with complex geometries can be coated using CMOS-compatible temperatures at and below 400 °C. The nozzle is placed about 30 cm away from the substrate, ensuring that the droplets have no horizontal velocity when reaching the vicinity of the substrate. The long distance between nozzle and wafer also ensures that all large liquid droplets will be eliminated prior to reaching the substrate, ensuring a uniformity in the droplet size distribution and, thereby, a uniformity in the deposited film.

The steps that describe the processes taking place during spray pyrolysis deposition are [58]:

1. Atomization of the precursor solution;
2. Aerosol transport of the droplet;
3. Decomposition of the precursor to initiate film growth.

In this section, the dependence of the deposition time and temperature on the film thickness is described, and an empirical model for the growth of tin oxide films is introduced. In addition, the architecture of a SnO_2_ sensor is presented, and the model is implemented in order to generate a full sensor structure, useful for further simulation studies.

### 4.1. Model for Spray Pyrolysis Deposition

**Figure 16:**
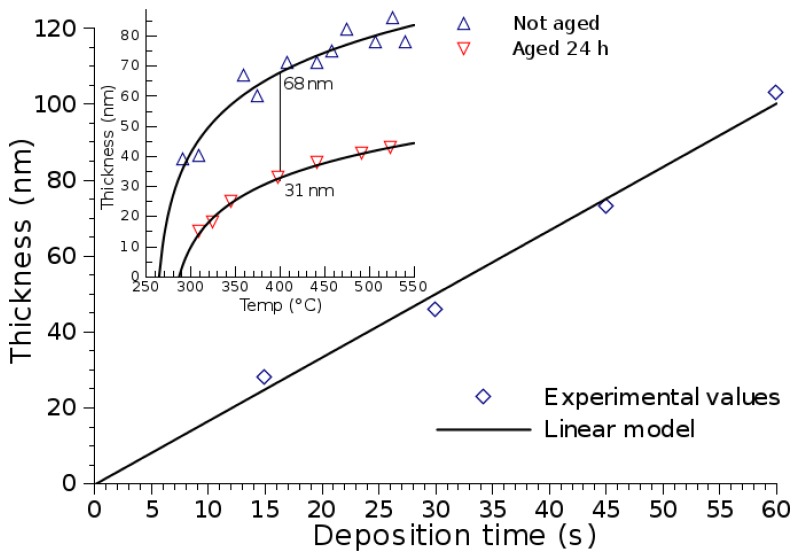
Deposited SnO_2_ thickness *versus* deposition time. The inset shows the dependence of temperature and solution aging on the deposited thickness. A half-day aged solution is used in the given experiment.

Several factors influence the film growth process, such as the distance between the nozzle and substrate, the length of the precursor solution aging, the air pressure, the solution volume, the temperature and the deposition time [4]. For the presented model, the ambient pressure, the nozzle distance to the substrate and the solution aging remain constant. In Figure 16, the dependence of the film thickness on the deposition time is shown, when the processing temperature is set to 400 °C. A linear relationship is evident, while a logarithmic dependence on the wafer temperature has also been observed [58]. An Arrhenius expression that best describes the deposition time (*t*, in seconds) and temperature (*T*, in Kelvin) dependence on the SnO_2_ thickness (*d*, in μm) is given by:

(6) $$d\left(t,T\right)=A\cdot t\cdot e^{-E/k_{B}T}$$

where *A* = 3.1 μm/s and *E* = 0.427 eV. A 50-nm thickness is reached after spray pyrolysis is performed for a 30-second burst at 400 °C. The inset of Figure 16 suggests that, depending on the solution aging, a 31-nm to 68-nm thickness is to be expected [4]. With a half-day aged solution, a 50-nm thickness is obtained under the same process conditions. When the technique is used to deposit a thin film on a step structure, no significant variation in the film thickness is noted [59]. This fact, combined with the high uniformity of the deposited film, means that the deposition occurs after the droplet approaches the heated substrate and forms a vapor. The droplets do not appear to directly impact the substrate surface in liquid form.

### 4.2. Spray Pyrolysis Deposition on Gas Sensor Geometries

**Figure 17:**
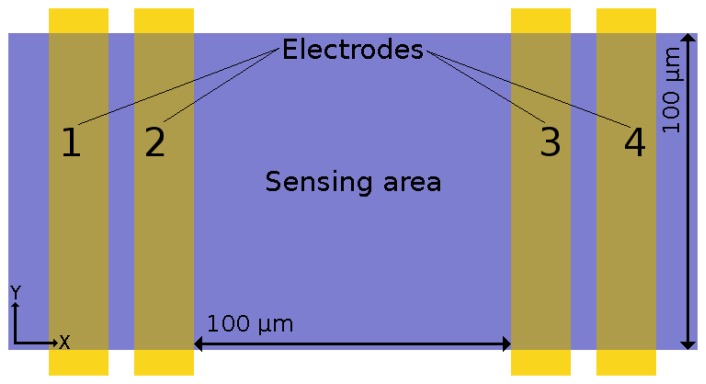
Top view of the electrode locations on the substrate. The sensing area (SnO_2_) is deposited on top of the electrodes. The sensing area is 100 μm × 100 μm.

The sensing layer of a gas sensor is deposited on top of a substrate, which is provided with four electrodes, as shown in Figure 17. The full structure is placed on top of an electrically-insulating layer, which separates the substrate from a heater placed below it.

**Figure 18:**
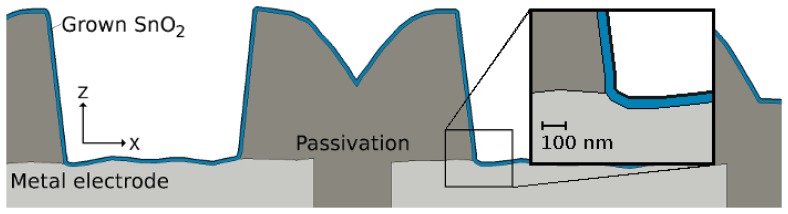
Geometry of the deposited SnO_2_ on the aluminum electrodes and silicon oxide passivation layers.

A two-dimensional view of a cut through the sensor geometry, including the electrode metals and passivation layer, is shown in Figure 18. The tin oxide deposition on the trench and step structure is modeled with Equation (6). The simulation of the deposition on complex geometries is performed using a Monte Carlo method within a level set framework [60]. A single particle species is considered during the deposition. As the simulation is initiated, multiple particles are generated with a flux in the direction of the substrate. Each particle is described by its arriving direction and energy, as well as its reflected direction and energy, should the particle not stick to its initial point of contact. It was found that a sticking coefficient of 0.01 has the best fit to experimentally-observed data. Figure 18 shows the resulting structure, when the spray pyrolysis deposition is simulated at 400 °C for 30 seconds. A uniform 50-nm SnO_2_ film covers the passivation and electrode layers.

For a 50 nm-thick SnO_2_ layer, deposited using the spray pyrolysis technique, the X-ray diffraction pattern is given in [61]. When the deposition is performed at 400 °C, as was done in this study, the material exhibits polycrystalline peaks characteristic of the cassiterite phase [61]. The predominant reflections are from the crystallographic (110) and (200) planes, parallel to the substrate. In addition, small peaks are noted from other main planes of cassiterite (211), (101) or (211), indicating that the film has texture.

### 4.3. Gas Sensing Capability of the Sprayed SnO_2_ Film

**Figure 19:**
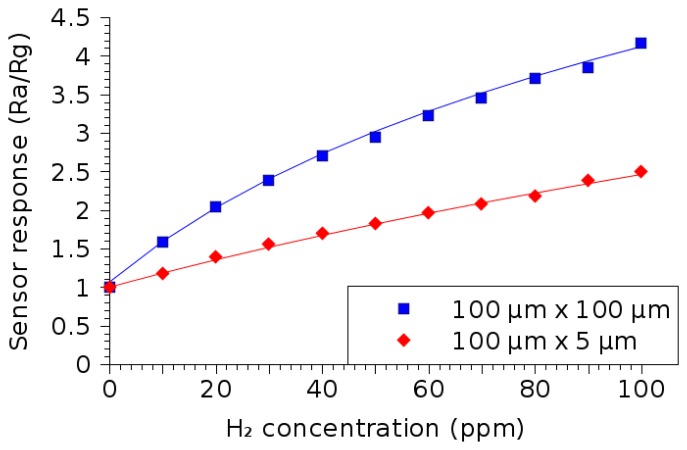
SnO_2_ thin film sensor response dependence on the concentration of hydrogen in the ambient gas. The 50-nm thin film is deposited using a spray pyrolysis burst of 30 s, while the wafer is heated to 400 °C. The sensing measurements are performed at 350 °C.

Two sensor structures have been used in order to test the sensor's response in the presence of H_2_ at a 350 °C temperature [16]. The dimensions of the two structures are 100 μm × 100 μm and 100 μm × 5 μm, respectively. A 50-nm layer of SnO_2_ has been deposited using spray pyrolysis, as described in the previous section. The effect of the presence of H_2_ in the atmosphere on the resistive response of the SnO_2_ layer is shown in Figure 19. It is evident that the sensor can detect the presence of hydrogen in the atmosphere down to 10 ppm. The symbols in the figure represent the measured sensor responses, while the solid lines represent the best-fit lines, the equations of which are given in Table 7.

## 5. Summary and Conclusions

Smart gas sensors that can detect harmful and toxic gases in the environment have recently grown in popularity and research interest. While many CMOS and MEMS devices have experienced extreme miniaturization, gas sensors are still lagging in the form of bulky, power-intensive structures. The capability of metal oxides, such as ZnO, In_2_O_3_, ITO and SnO_2_, to detect various gases when heated to temperatures between 250 °C and 550 °C has recently been exploited in order to attempt to introduce gas sensors fit for wearable technologies. At these increased temperatures, the metal oxide material adsorbs oxygen at the surface, causing a depletion layer to form in the thin film. The result is an overall decrease or increase in the film resistance, depending on whether the material is n-type or p-type, respectively. When a target gas is introduced, it reacts with some of the surface oxygen, removing them from the film and thereby reducing the depletion layer and the film resistance. The difference between the resistance in air and the resistance in the presence of the reacting gas can then be exploited as the sensor response. ZnO, In_2_O_3_, ITO and SnO_2_ thin films have been examined for their ability to detect the presence of liquid petroleum gas, hydrogen, ethanol, acetone, carbon monoxide and nitrogen dioxide in the environment. The sensing reaction has a general logarithmic trend, which tends to saturate as more reacting gas interacts with the surface oxygen.

The stress generation during the deposition of metal oxides has also been examined. The post-processing stress is a combination of the intrinsic stress, which develops during the Volmer–Weber film growth, and the thermo-mechanical stress, which develops as a result of material cooling to room temperature from a thermal deposition step. The surface stress values for the analyzed films have been derived, and their dependence on the contact angle between the island surface and the substrate has been shown. The thermo-mechanical stress has been analyzed using finite element methods, and a linear relationship between the process temperature and the stress has been demonstrated.

Spray pyrolysis deposition has been introduced in order to generate a SnO_2_ sensor and to test its performance. A model for spray pyrolysis has been presented and implemented within a level set topography simulator. The film thickness shows a linear relationship with spray time and a logarithmic relationship with the process temperature. The generated sensor structure can be successfully used for the detection of hydrogen in the atmosphere down to 10 ppm.

## Figures and Tables

**Table 1:** Summary of the gas sensing properties of ZnO, shown in Figure 6. The equation column shows the best-fit lines for the sensitivity of the thin film (solid lines in Figure 6).

**Reacting gas**	**Temperature**	**Equation**	**Concentration**
Hydrogen (*H*_2_) [28]	350 °C	4.43 · *e*^0.0088·*C_ppm_*^	50 ppm–400 ppm

Liquid petroleum gas (LPG) [29]	300 °C	43.1 · *e*^0.0063·*C_ppm_*^	20 ppm–100 ppm

Acetone ((CH_3_)_2_CO) [30]	275 °C	382 · *ln*(*C_ppm_* + 1686) – 2737	400 ppm–4000 ppm
	300 °C	710 · *ln*(*C_ppm_* + 2705) – 5561	

Ethanol (C_2_H_5_OH) [30]	275 °C	157 · *ln*(*C_ppm_* + 385) – 850	
	300 °C	290 · *ln*(*C_ppm_* + 2330) – 2100	

**Table 2:** Summary of the gas sensing properties of In_2_O_3_, shown in Figure 7. The equation column shows the best-fit lines for the sensitivity of the thin film (solid lines in Figure 7).

**Reacting Gas**	**Temperature**	**Equation**	**Concentration**
Ethanol (C_2_H_5_OH) [33]	250 °C	74 · *ln*(*C_ppm_* + 21) – 230	50 ppm–2000 ppm
Liquid petroleum gas(LPG) [33]	250 °C	33 · *ln*(*C_ppm_* + 44.4) – 125	

Carbon monoxide (CO) [34]	300 °C	246 · *ln*(*C_ppm_* + 1797) – 1848	150 ppm–4000 ppm
Hydrogen (*H*_2_) [34]	300 °C	11.6 · *ln*(*C_ppm_* + 435) – 72	

**Table 3:** Summary of the gas sensing properties of ITO, shown in Figure 8. The equation column shows the best-fit lines for the sensitivity of the thin film (solid lines in Figure 8).

**Reacting Gas**	**Temperature**	**Equation**	**Concentration**
Ethanol (C_2_H_5_OH) [35]	350 °C	17.7 · *ln*(*C_ppm_* + 162.6) – 90.2	<600 ppm
Acetone ((CH_3_)_2_CO) [35]	350 °C	5.14 · *ln*(*C_ppm_* + 17.4) – 14.5	
Nitrogen dioxide (NO_2_) [35]	280 °C	18.5 · *ln*(*C_ppm_* + 250) – 102	

**Table 4:** Summary of the gas sensing properties of SnO_2_, shown in Figure 9. The equation column shows the best-fit lines for the sensitivity of the thin film (solid lines in Figure 9).

**Reacting gas**	**Temperature**	**Equation**	**Concentration**
Carbon monoxide (CO)	287 °C [38]	*e*^0.00276·*C_ppm_*^	< 150 ppm
		0.37 · *ln*(*c_ppm_*) – 0.35	150 ppm–500 ppm

	400 °C [39]	9.9 · *ln* (*C_ppm_* + 79) – 42.2	< 500 ppm

Hydrogen (H_2_) [16]	350 °C	2.58 · *ln*(*C_ppm_* + 44) – 8.74	< 100 ppm

**Table 5:** Properties of metal oxides ZnO, In_2_O_3_, indium-tin-oxide (ITO) and SnO_2_, required for the simulation of stress evolution during Volmer–Weber growth and grain coalescence. CTE, coefficients of thermal expansion.

**Characteristic**	**ZnO**	**In_2_O_3_**	**ITO**	**SnO_2_**
Young's modulus *E* (GPa)	210 [40]	145 [41]	116 [42]	253 [43]
Poisson's ratio *ν*	0.36 [44]	0.31 [45]	0.33 [46]	0.293 [43]
CTE *α* (K^−1^)	3.9 × 10 ^−6^ [37]	6.7 × 10^−6^ [37]	8.5 × 10^−6^ [47]	4.0 × 10^−6^[37]
Density *ρ* (g/cm^3^)	5.67 [37]	7.12 [37]	7.18 [52]	6.99 [37]
Average grain size *D* (nm)	13–16.5 [49]	10–80 [53,54]	10–40 [55]	12–16.5 [56]
Melting point (°C)	2240 [37]	2190 [37]	1913 [52]	1900 [37]
Intrinsic stress *σ_i_* (GPa)	2.3–3.43 [48,49]	0.4–1.5 [50]	2.1–2.3 [42]	1.5 [51]

**Table 6:** Surface stress for the metal oxide materials introduced in this study. The average grain radius and island contact angle with the substrate are set to *r* = 15 nm and *θ* = 50°, respectively.

**Material:**	**ZnO**	**In_2_O_3_**	**ITO**	**SnO_2_**
**Intrinsic stress** *σ_i_*	2.3 GPa	1.0 GPa	2.1 GPa	1.5 GPa
**Surface stress** *f*	21 J/m^2^	9 J/m^2^	19 J/m^2^	14 J/m^2^

**Table 7:** Equations corresponding to the best-fit curves for the measured data plotted in Figure 19. The sensor response displays a logarithmic dependence on the H_2_ concentration, as was previously noted in Section 2.

**Dimensions**	100 μm × 100 μm	100 μm × 5 μm
**Equation**	2.58 · *ln*(*C_ppm_* + 44.2) − 8.7	3.03 · *ln* (*C_ppm_* + 161.5) − 14.4
